# Superelasticity in Shape Memory Alloys—Experimental and Numerical Investigations of the Clamping Effect

**DOI:** 10.3390/ma18143333

**Published:** 2025-07-15

**Authors:** Jakub Bryła, Adam Martowicz

**Affiliations:** Department of Robotics and Mechatronics, Faculty of Mechanical Engineering and Robotics, AGH University of Krakow, al. A. Mickiewicza 30, 30-059 Krakow, Poland; jakbryla@agh.edu.pl

**Keywords:** shape memory alloys, superelasticity, clamping effect, boundary conditions, nonlinear material properties, thermovision

## Abstract

Loading and clamping schemes significantly influence the behavior of shape memory alloys, specifically, the course of their solid-state transformations. This paper presents experimental and numerical findings regarding the nonlinear response of samples of the above-mentioned type of smart materials observed during tensile tests. Hysteretic properties were studied to elucidate the superelastic behavior of the tested and modeled samples. The conducted tensile tests considered two configurations of grips, i.e., the standard one, where the jaws transversely clamp a specimen, and the customized bollard grip solution, which the authors developed to reduce local stress concentration in a specimen. The characteristic impact of the boundary conditions on the solid phase transformation in shape memory alloys, present due to the specific clamping scheme, was studied using a thermal camera and extensometer. Martensitic transformation and the plateau region in the nonlinear stress–strain characteristics were observed. The results of the numerical simulation converged to the experimental outcomes. This study explains the complex nature of the phase changes in shape memory alloys under specific boundary conditions induced by a given clamping scheme. In particular, variation in the martensitic transformation course is identified as resulting from the stress distribution observed in the specimen’s clamping area.

## 1. Introduction

Smart materials (functional materials) continuously gain increasing scientific and engineering interest due to their advantageous properties [1]. Amongst all smart materials, there are various distinguished types of shape memory materials (SMMs), including shape memory alloys (SMAs) [2] and shape memory polymers (SMPs) [3]. The significant worldwide economic impact of SMMs motivates the continuous research and interest of many industrial sectors in improving the properties and usability of such smart materials. As reported in [4], the global SMM market in 2024 was USD 16.88 billion, and is estimated to be USD 36.72 billion by 2032. SMMs are greatly applied in high-performance areas, e.g., automobiles, aerospace, robots, and biomedical devices, enabling minimally invasive surgery. On one hand, SMAs advantageously become more and more biocompatible and flexible. Complementarily, SMPs are widely used in wearable electronics, adaptive textiles, and smart infrastructure. Many innovative and customized products are presently brought to the market thanks to the rapid improvement in SMM properties. The nickel–titanium (NiTi) SMA, known as nitinol, remains of particular interest. This alloy became the first SMA engineering material, and its shape memory effect was discovered at the Naval Ordnance Laboratory in 1959. Its unexpected discovery has successfully contributed to establishing a new group of smart materials.

The specific property of interest of SMAs, particularly nitinol, is their capability to remember geometric shapes and withstand excessive elastic deformations. In other words, the functionality mentioned above indicates the material’s transformation back to its primary shape under an external mechanical or thermal load. The shape memory effect in SMAs is related to solid phase transformation, which appears under temperature changes. Moreover, changing the internal structure of a material leads to the latter of the two above-referenced unique behaviors. Hence, following the macroscopic response of an SMA material, three phenomena are distinguished: the one-way memory effect, the two-way memory effect, and superelasticity (pseudoelasticity). The scope of the present paper relates to superelasticity. Therefore, the authors only address this phenomenon via experimental and computational investigations. More detailed information about the other phenomena in SMAs can be found in the literature [2,5].

**Figure 1:**
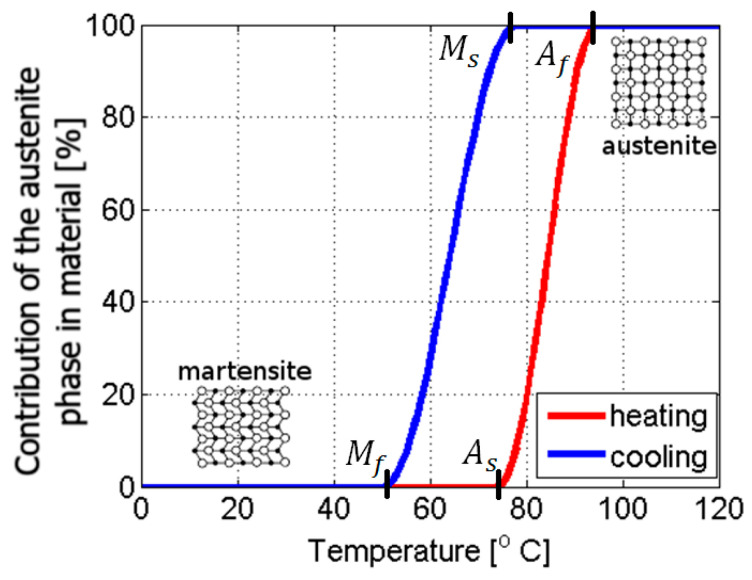
The percentage contribution of the austenite phase in an SMA material in relation to temperature [2,6].

SMAs are primarily composed of martensite and austenite phases. Volume fractions of these phases are related to the material’s temperature and stress fields. Below, the thermal relation in SMAs is briefly discussed. The austenite phase exists at higher temperatures than martensite does. The approximated percentage contribution for the austenite phase concerning temperature is presented in Figure 1 [2,6].

**Figure 2:**
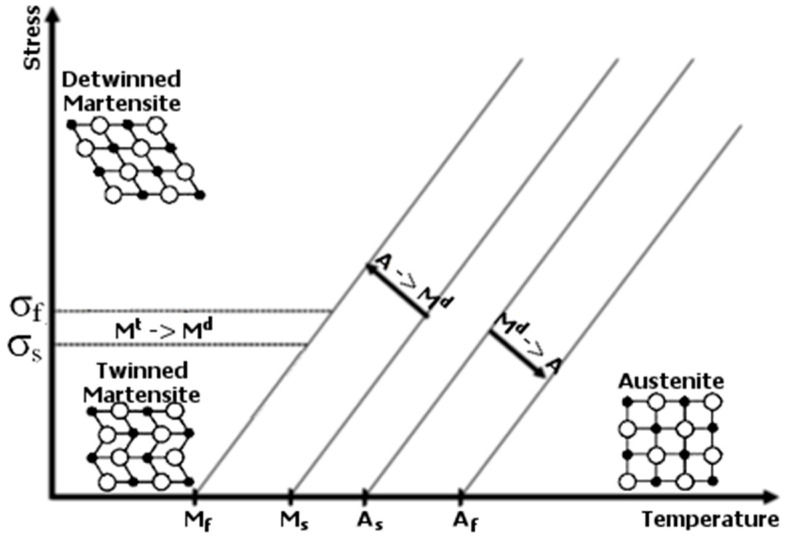
SMA phase diagram [2].

When studying Figure 1, one can notice four characteristic temperatures crucial for the material’s properties: *M_s_*, *M_f_*, *A_s_*, and *A_f_*. These quantities declare the initiation and completing conditions for bi-directorial thermally induced solid phase transformations, where *M_s_* is the starting temperature of forward transformation, which is the change from austenite to martensite, *M_f_* is the finishing temperature of forward transformation, *A_s_* is the starting temperature of reverse transformation, which is the change from martensite to austenite, and *A_f_* is the finishing temperature of reverse transformation. The forward and reverse transformations create the phenomenon called martensitic transformation. Variation in the volume fractions of the contributing phases is essential due to its influence on the resultant material properties, such as stiffness, electric resistivity, and others [7,8,9]. The crystalline structure of martensite enables more significant deformations compared to austenite, which is schematically presented in Figure 1. The characteristic temperatures of phase transformations can be experimentally identified during a differential scanning calorimetry (DSC) test. The DSC results show that a portion of thermal energy is released from a material during forward transformation. Accordingly, some energy is absorbed during reverse transformation [10,11]. It is also worth noting that the four characteristic temperatures above, *M_s_*, *M_f_*, *A_s_*, and *A_f_*, increase following stress growth in an SMA material. The relations between these temperatures and stress are often assumed to be linear, exhibiting satisfactory convergence to the observations made during experiments [5]. A representative diagram of the dependency between the transformation temperatures and stress is presented in Figure 2.

The superelasticity effect is related to the stress–temperature relation illustrated in Figure 2. Above the temperature *A_f_*, an SMA sample only consists of the austenite phase. However, an external load applied to the material generates a non-zero stress state. As stated before, the increase in stress impacts the values of the characteristic temperatures. If the mechanical load is sufficiently large, then the temperature *M_s_* at a given stress state can be high enough—concerning its nominal counterpart, which is considered the material property—to initiate forward transformation. The change from the austenite phase to the martensite phase under a given load generates a reversible deformation greater than that observed for a material not exhibiting phase transformation. The described phenomenon causes a considerable elastic strain—even at the level of 8%, which is much beyond the capabilities of regular construction materials, e.g., steel, aluminum, and titanium. The plateau region observed in the hysteretic stress–strain relations and the material’s damping ability, amongst others, are the reasons for the vast application range of SMAs. These two features have already been studied by the present work’s authors [12]. Another crucial issue is the variation in the course of phase transformation during cyclic loading of SMAs. The authors of the work [13] successfully validated the proposed numerical model to accurately track the respective change in solid phase volume fractions. Complementarily, the electrical resistance change in NiTi wires was experimentally identified in the presence of cyclic stretching and the induced reorientation of material structure [14].

There are known concepts, computational simulations, and experimental investigations employing SMAs to improve the dynamic response in various types of constructions and devices, considering both thermally activated shape memory effects and superelasticity, as reported in the works [15,16]. These recent studies also introduce the formulation of nonlocal elasticity via peridynamics to SMA models. The proposed approach sounds promising as it allows for convenient and effective handling of the nonlinearities involved in the material governing equation. The capability of controlling the structural stiffness, achieved with SMA components, provides a novel means to assure the desired operational stability, improvement in dynamic properties, and mechanical response [15]. Therefore, SMAs may act as active or passive solid components to modify the effective stiffness or reduce vibrations via energy dissipation [12], simultaneously avoiding the necessity of introducing any classical linear or rotary joint. SMAs are used in many devices as solid actuators dedicated to various systems with movable joints. Hence, SMAs are often and eagerly used in projects implemented in aerospace, spacecraft engineering [17,18], and robotics [19,20,21]. SMAs, particularly nitinol, are also very promising for medical applications because of their biocompatibility [22,23,24]. However, it should be noted that additional requirements regarding the use of SMAs must be met in this case due to the direct impact on human lives.

Even though SMAs have found many interesting applications, the surprising fact is that until now, no appropriate standard has been published to assure safe and effective solutions based on SMA components. The only standards that address SMMs are the ASTM norms. General information about the materials is provided in [25], while the medical restrictions are covered in [26]. Some standards show methods to estimate material properties. The standard in [27] references the determination of the temperatures *A_s_* and *A_f_*. The standard in [28] describes experimental tests to estimate other material properties, such as lower and upper plateau strength, tensile strength, and residual elongation. Moreover, the cited standard notes that the methods used to determine Young’s modulus are beyond the document’s scope. There is only mention of the limitation of strain speed due to the exothermic and endothermic nature of the martensitic transformation [29]. Additional suggestions should be taken into account from the standards in [30,31], of which the latter is more suitable for SMAs [32]. Complementarily, the nanotechnological approaches originating from soft matter characterization may also be used to more comprehensively identify material properties, including Young’s modulus [33]. As described in the cited study, various experimental frameworks can extract elastic moduli with the aid of atomic force microscopy. Moreover, [34] reports that various deterministic and stochastic methods may successfully enhance the tensile testing procedure. As confirmed, the soft computing approach may effectively improve the design of experiments and, in turn, increase the quality of inference about material properties based on the registered stress–strain curves. Nonetheless, the complexity of the above-described methods of elastic modulus identification often justifies the standard use of a fatigue testing machine to estimate the value of Young’s modulus in a classical way, which may be a sufficient approach.

At this point, it is essential to note that there exist works in which the test equipment used for SMAs do not meet the standard’s requirements [2]. On the other hand, some papers study the influence of the strain rate and absorption/release of thermal energy on SMA response during tensile experiments [32,35]. However, the present paper refers to the less-known fact described in the works [36,37]. The cited works study the influence of stress concentration due to the gripper effect on the material’s response recorded during tensile tests. The referential case for the present work was formulated based on the results obtained from standard tensile tests, which used typical grips clamping an SMA sample.

The phenomenon of superelasticity in nitinol is addressed explicitly in the current work. The overall motivation of the present study is to take advantage of the nonlinear characterization of SMA samples, i.e., their hysteretic behavior, to model and investigate the clamping effect. This work should fill the knowledge gap regarding reliable modeling of the course of solid phase transformations observed within the body of an SMA specimen undergoing mechanical load [38]. This paper consists of six sections. After introductory Section 1, Section 2 discusses the experimental results obtained for the referential case study. Next, a description and the results of the numerical simulation carried out for the referential experimental configuration are presented in Section 3. Section 4 describes tensile tests with the additional use of thermal cameras. The final configuration of the experimental stand equipped with dedicated grips and the obtained results are presented in Section 5. It is worth noting that in the three considered experimental cases described in Section 2, Section 4, and Section 5, the separate tested samples were extracted from the same original wire coil. As a result, the samples of the same material properties that had not been previously stretched could undergo cyclic mechanical loading. A summary of the findings and the authors’ conclusions are provided in Section 6.

## 2. Superelasticity—Tensile Test

**Figure 3:**
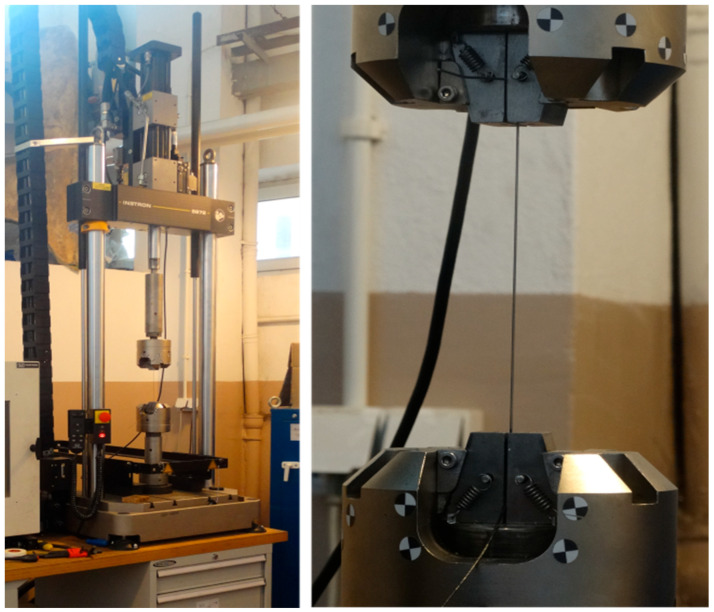
The experimental stand, on the left, is the fatigue testing machine Instron8872, with an SMA wire mounted in the grips.

According to the referential papers listed in Section 1, the authors of the present work performed tensile tests. A nitinol wire acquired from the company Smartwires (Brno, Czech Republic, https://smartwires.eu) was used. The properties of the tested wire are as follows: a diameter of 1 mm and an activation temperature equal to 10 °C ± 5 °C. Consequently, the nitinol wire exhibits the superelastic effect at room temperature. Load generation and data acquisition were carried out by using the fatigue testing machine Instron8872 with a maximum load of 10 kN. The experimental stand is shown in Figure 3.

Four experiments were performed for a nitinol wire sample of a length equal to 133 mm. A test schedule was designed to observe the superelasticity effect. The consecutive experimental steps for each testing case are presented in Table 1.

The tension of the wire was controlled by the displacement rate resulting from the superelasticity phenomenon. Low deformation rates allow for the neglect of the influence of exothermic and endothermic phenomena observed during phase transformations in an SMA sample [10,11]. Additionally, using the displacement as a control parameter allows us to directly monitor the strain in the tested material, which should not exceed the level of 8%.

**Figure 4:**
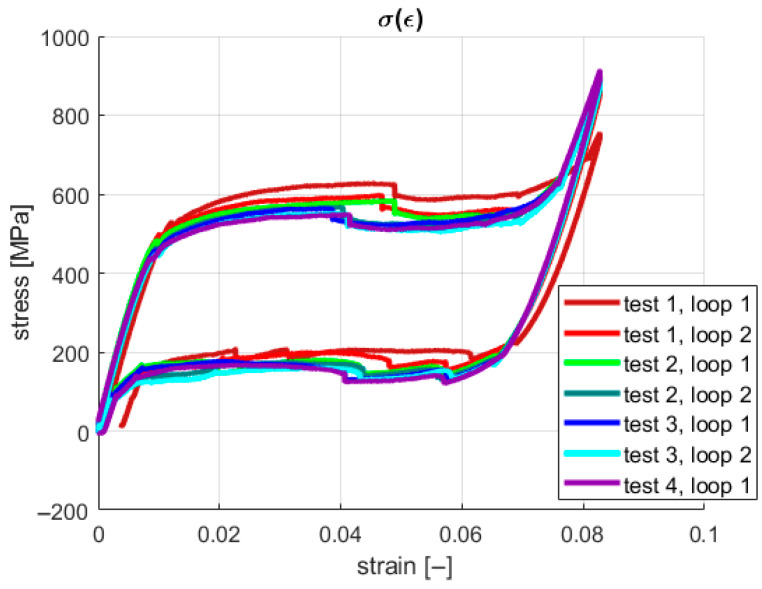
Stress–strain characteristics from tensile tests.

**Figure 5:**
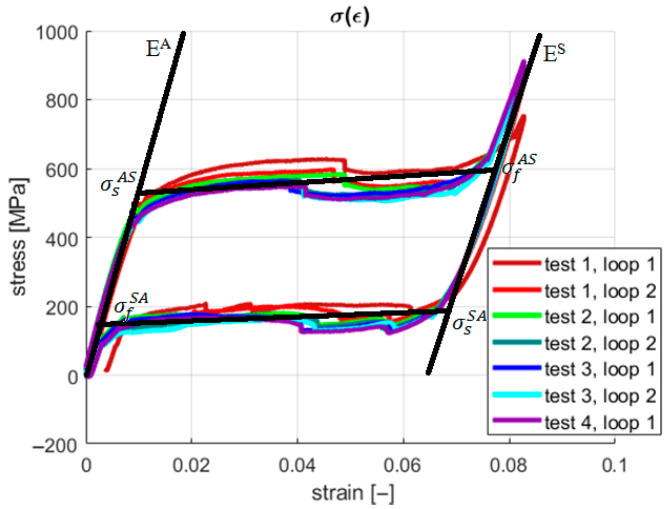
The main properties of the SMA material based on the stress–strain characteristics.

The acquisition system saved information about the grip displacement and generated force at a given sampling frequency. However, the introduction of the geometric parameters of the sample provided the possibility of presenting the results in a more desired and readable way, using a stress–strain relationship. The characteristics for all four tests are shown in Figure 4 and Figure 5. During the described experiments, the sampling frequency was equal to 1 kHz.

It is shown in Figure 4 and Figure 5 that under the first loading cycle, the sample gives a different response compared to the consecutive cases. This phenomenon is common and relates to the gradual cancellation of the initial looseness revealed after the installation of the wire in the testing machine grips. Therefore, the authors analyzed only the succeeding six cycles. Tensile tests were performed to obtain information about the material’s properties. Young’s modulus for both phases and the four characteristic stresses were investigated in this case. The first approximation of Young’s modulus was found by calculating the tangents on the stress–strain characteristics (Figure 5). Next, these values were used to develop a numerical model as an initial reference. The identified material parameters are presented in Table 2.

## 3. Simulation of the Tensile Test

**Figure 6:**
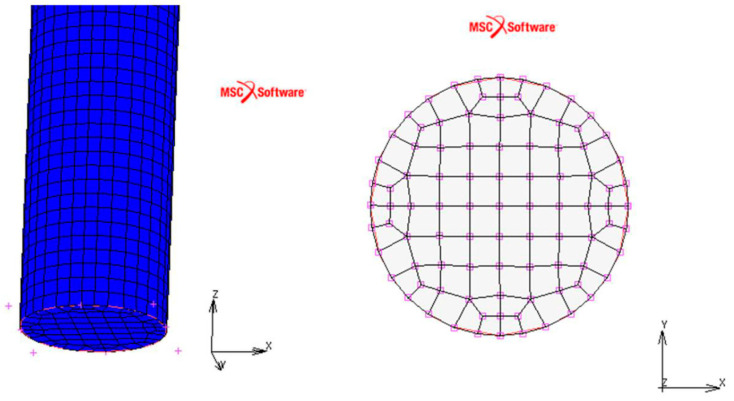
FE model of SMA wire.

Numerical simulations were performed in MSC.Software/Marc ver. 2018 environment using the finite element (FE) method. The authors described the model in their previous paper [37]. Comprehensive information regarding SMA modeling can be found in [39]. The model of the wire sample was created using three-dimensional (3D) FEs, which is purposeful according to a complex spatial stress field observed during phase transformation in an SMA material. The model consists of 101,080 FEs to meet the geometric requirements of the cubic elements—ensuring the same length for each cube edge. The FE model of the investigated SMA sample is shown in Figure 6.

**Figure 7:**
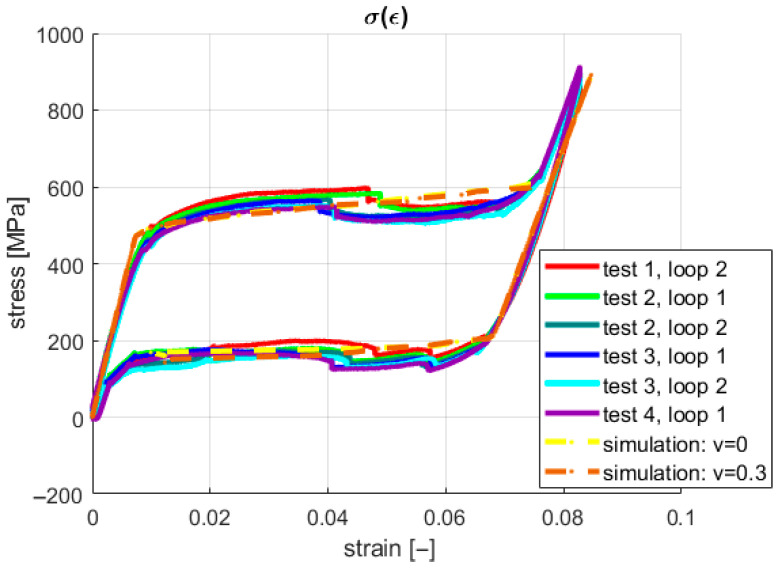
A comparison of the experimental and simulation results: the stress–strain characteristics.

The material properties declared in the numerical model are listed in Table 2 in Section 2. Two types of analyses were carried out, namely for the three-dimensional and one-dimensional (1D) stress field, where, respectively, Poisson’s ratios were ν = 0.3 and ν = 0. The results for both experimental tests and simulations are shown in Figure 7.

The characteristics shown in Figure 7 present the convergence between the simulation and experimental results. However, comparing the material properties evaluated based on the experiments (Figure 5) and those being considered in the simulations (both found in Table 2 in Section 2) shows some discrepancy. Greater values of the characteristic stresses were defined in MSC.Software/Marc software ver. 2018 concerning the values identified based on the experiments to assure similarity between the final shapes of the stress–strain characteristics for both simulations and real tests. Furthermore, the difference between the 1D and 3D stress field results was identified as negligible. Based on the above statements, the 3D stress was assumed not to significantly impact the strain field in a 1D tensile test. In both cases, however, for a zero and non-zero Poisson’s ratio, the stress generated by 1D elongation was underestimated concerning the displacement observed in the tests. Therefore, an additional source of the stresses in the sample should be identified to clarify the mentioned elongation underestimation. This paper’s main objective is to verify that the additional stresses in an SMA sample originate from the forces generated during grip compression. Considering the initial simulation studies presented in [37], in the present work, an attempt to experimentally confirm the above-stated thesis is performed by observating of thermodynamic processes in the material during phase transformations.

## 4. Tensile Test with Thermovision Monitoring

Martensitic transformation involves changing the material properties, e.g., Young’s modulus, resistivity, and thermal conductivity. Thanks to those changes, phase transformation can be identified and observed during experiments. One of the first methods applied to observe and track the course of martensitic transformation considers the resistivity variation of a sample [7,40]. In this approach, the percentage volume of the phases in a material may be effectively estimated. The second approach to phase change identification is related to the exothermic and endothermic nature of martensitic transformation. The latent heat’s release and absorption can be observed using a DSC test.

**Figure 8:**
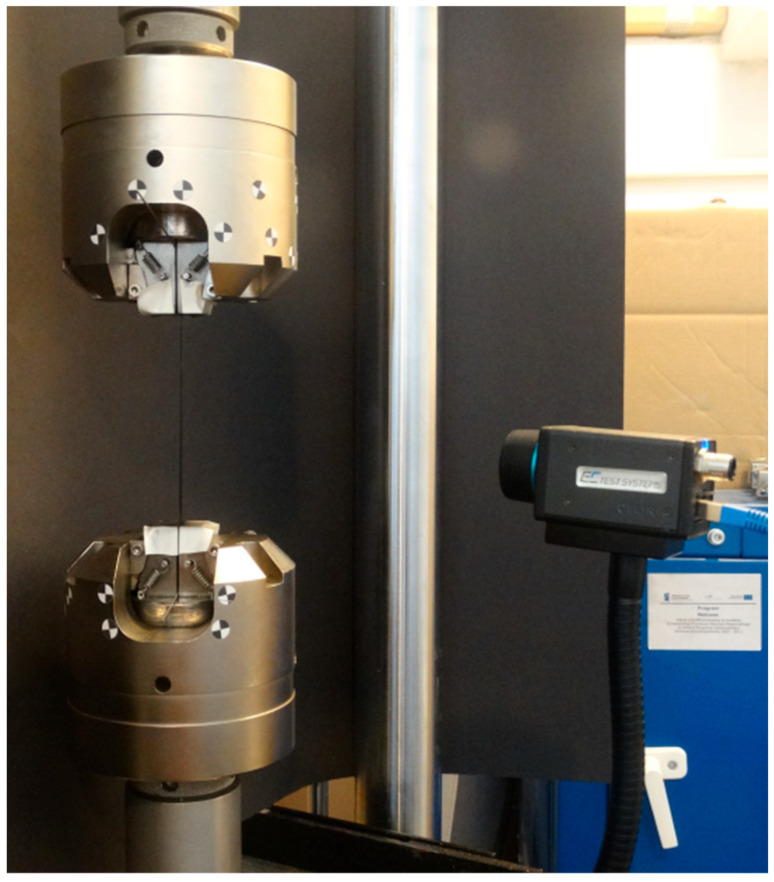
The experimental stand. The use of the thermal camera FLIR A35 allows us to observe thermal energy exchange during the phase transformation.

DSC tests can be used only to estimate the thermal properties of an SMA sample, e.g., transformation temperatures and thermal capacity. Thermovision-based techniques provide more possibilities regarding parameter identification which can be used during tensile tests [11]. It should be mentioned that resistance measurements allow for the estimation of the percentage volume of the phases in a material. In contrast, observations of temperature change only enable the assessment of process dynamics. In the present study, the authors aim to find a localization in a sample where the martensitic transformation occurs at a defined time. Therefore, thermovision is an appropriate method for this purpose. The elaborated test stand is shown in Figure 8.

**Figure 9:**
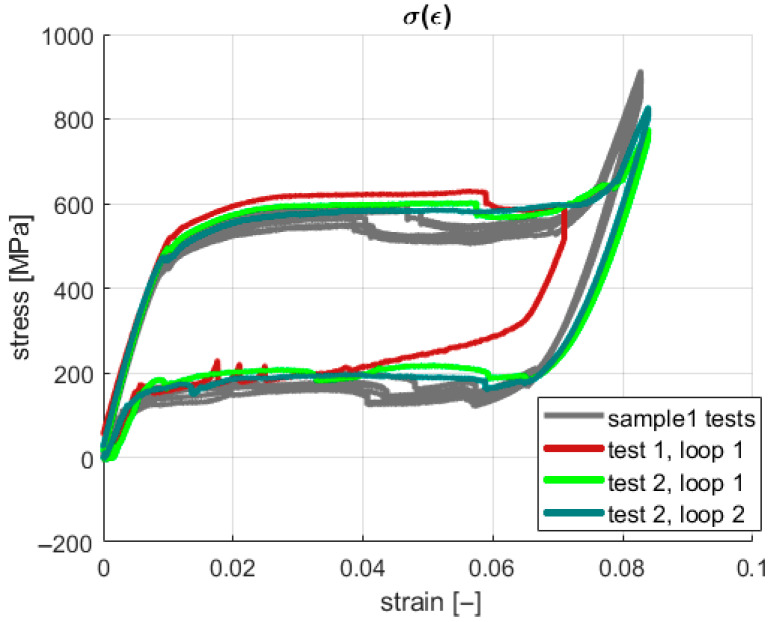
Experimental results for second SMA sample.

Taking advantage of thermal camera use, the experiments reported in Section 2 were repeated, considering additional thermal observations. Consequently, the second sample was made of the same type of material as the one previously used, with a length of 155 mm. The thermogram data were registered by the thermal camera FLIR A35. The experimental steps applied for all cases are presented in Table 3. The obtained results are presented in Figure 9.

According to the assumed agenda, the first test was conducted to cancel mechanical looseness. Hence, only the second test’s results, carried out within the two loops, are analyzed below. The tensile experiments for both samples give similar results. However, one can note that slight vertical shifts in the stresses and transformation strain characteristics are observed for the second wire.

**Figure 10:**
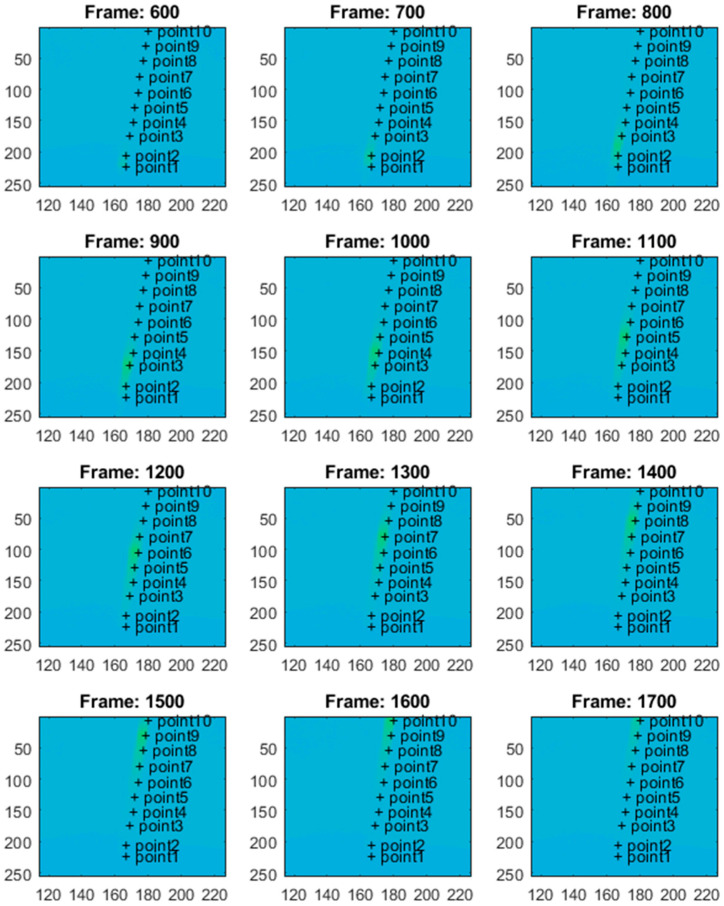
The selected points of the sample in the thermal camera views, marked with the plus signs. The moving green circles indicate the region of phase transformation during the tensile test.

**Figure 11:**
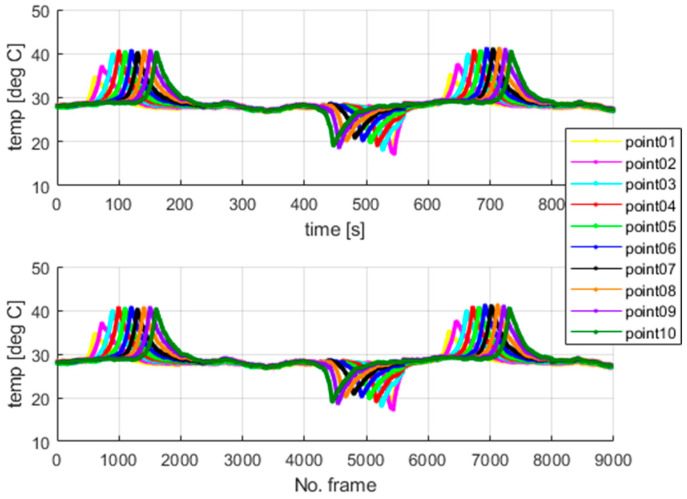
The temperatures measured at the points marked in Figure 10.

The thermal camera registered the results for the period of the first 900 s for the second test. The temperature change at the arbitrarily selected points within the examined sample is shown in Figure 10. Figure 10 presents the respective frames recorded by the thermal camera. The moving region of the wire, which is marked in green in the camera view, indicates the front of the gradually evolving martensitic transformation. This phenomenon originates from the accumulation of the temperature effects related to the release and absorption of latent heat. The point marked as “point1” is located near the grip, while the points with higher indexes are closer to the center of the wire. Temperature values for the selected points are presented in Figure 11.

**Figure 12:**
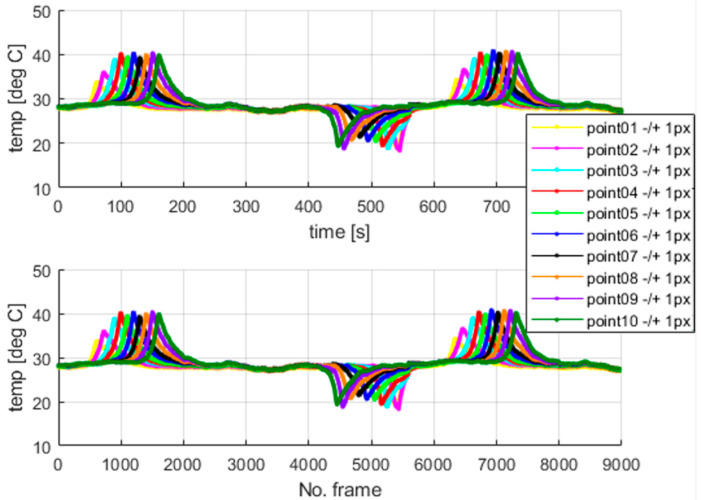
The average temperatures of areas of 3 × 3 pixels in the centers located at the points shown in Figure 10.

Additionally, the average temperatures for the arbitrarily sized areas were also calculated to avoid reflection errors. The auxiliary surfaces were set as squares of 3 × 3 pixels, where the points shown in Figure 10 mark the centers of those spots. The calculated average temperatures are shown in Figure 12. The camera sampling frequency was 10 Hz.

The highest values of the temperatures measured at the mentioned points are similar, which confirms the correctness of the undertaken data acquisition process. The results show that the martensitic transformation starts at the localization, where the wire stays in contact with the grips and evolves towards the wire’s center. The above observation also confirms the simulation results of the authors’ previous work [37]. The compression force generated by the grips causes higher local stresses in the SMA sample within the contact area. The martensitic transformation starts in the region where the stresses reach their characteristic value. Therefore, the way in which the wire was clamped, using standard grips, significantly changed the SMA response during tensile tests. This fact was observed in thermovision analysis, as shown in Figure 11 and Figure 12. Next, the authors performed additional tests to check the quantitative significance of the above-mentioned clamping phenomenon, which is reported in Section 5.

In the case of standard clamping jaws, sharp changes and piecewise monotonic characteristics for the temperature courses were observed even for the narrow averaging window of 3 × 3 pixels, as seen in Figure 12. Its use was sufficient to register the ongoing process of phase transformation. Similar shapes and amplitudes of the temporal temperature characteristics, which are shown in Figure 11 and Figure 12, confirm the homogeneous character of the course of the phase transformation front. The order of occurrence of the temperature maxima is maintained as the stress monotonically changes, i.e., increases or decreases. The temperature disturbance propagating at a constant speed is also clearly seen in Figure 10.

## 5. Superelasticity—Tensile Test with Dedicated Grips

**Figure 13:**
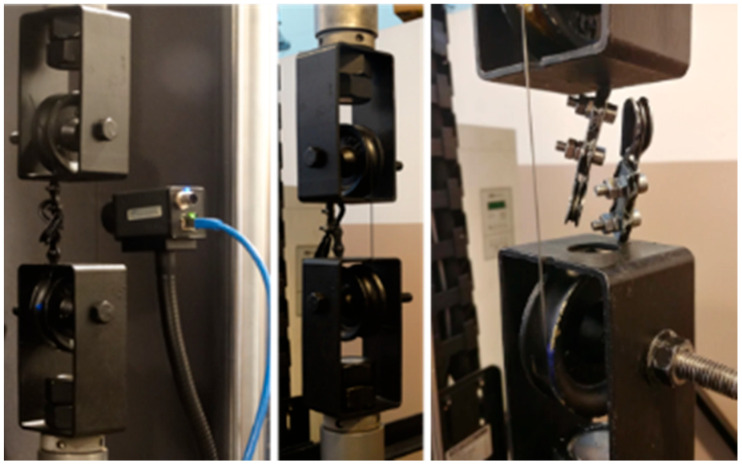
The unique construction of the fatigue testing machine grips dedicated to SMA samples.

Referring to the preliminary results, the authors carried out experiments to investigate the influence of the compression force generated by the fatigue testing machine jaws on an SMA sample’s response. The special, circular in shape, grips have been constructed. The experiment stand is shown in Figure 13. Additionally, an extensometer with a 25 mm long gauge was used for accurate measurements in the presence of a complex stress field along the grip cylinder. The constructed grips are proposed by the authors to significantly reduce contact-related stresses in the SMA sample. It is feasible due to eliminating the dual-side transversal compression load in classical clamping schemes. Hence, the expected stress gradients within the contact area are smaller compared to the case where the standard grips are installed, as presented in the previous Sections. The reduction in the stresses was assured by offsetting the fixation place on the other side of the grip cylinder. It is worth noting that the force generated between the jaws and the sample should not impact the test results if the cylinder radius is large enough. Therefore, several solutions for mounting a specimen may be used and tested. In this case, the wire was placed between two pinched plates so that the compressive forces counteracted any movement.

**Figure 14:**
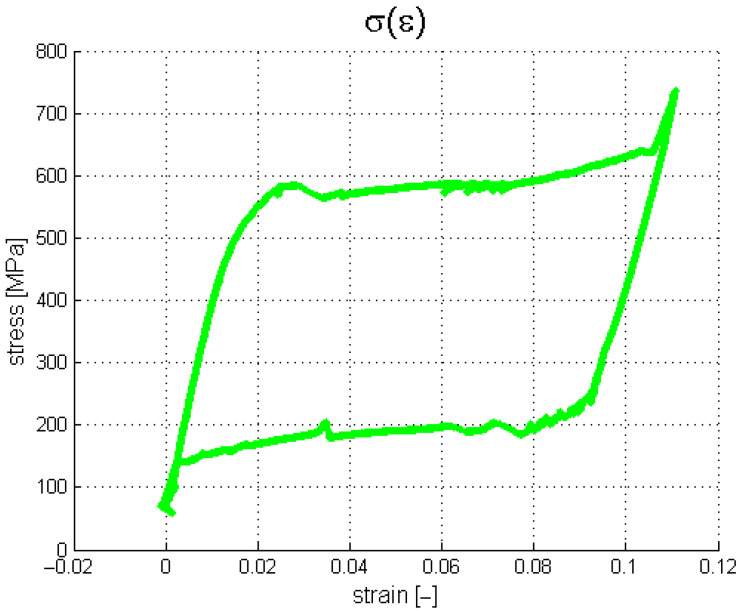
Experimental results for third SMA sample.

The tested sample was made of the same material as in the previous experiments. Its length was 165 mm. The experimental steps, which were the same for each case, are presented in Table 4. It should be noted that the load rate was changed from 0.05 mm/s up to 0.5 mm/s. This modification aimed to improve the resolution of the results obtained from the thermal camera. If an SMA sample releases or absorbs a specific portion of thermal energy over a shorter period, the temperature gradient increases. The results registered by the testing machine (stress) and the extensometer (strain) are presented in Figure 14.

**Figure 15:**
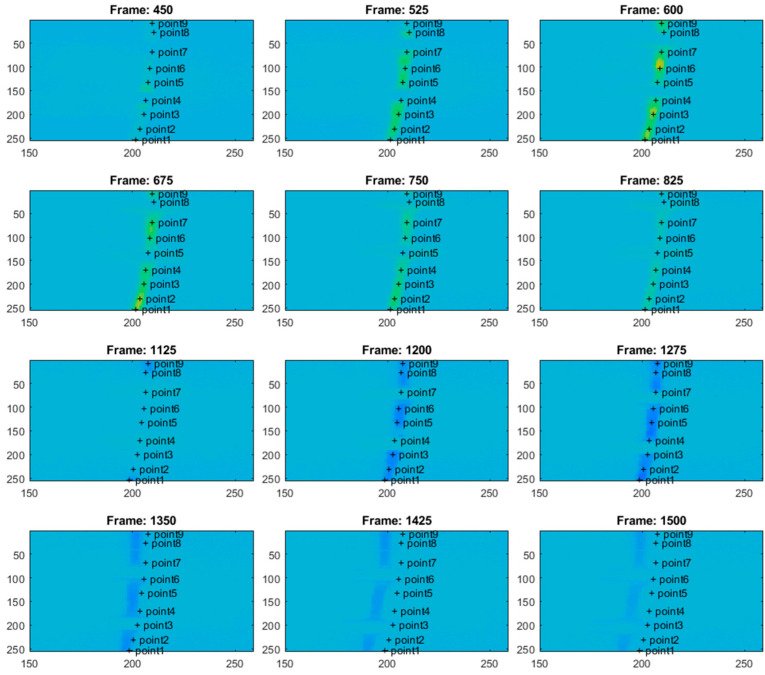
Selected points of the SMA sample in the thermal camera views, marked with the plus signs. In the presented case, a much more uniform phase transformation process is observed within the monitored part of the wire.

**Figure 16:**
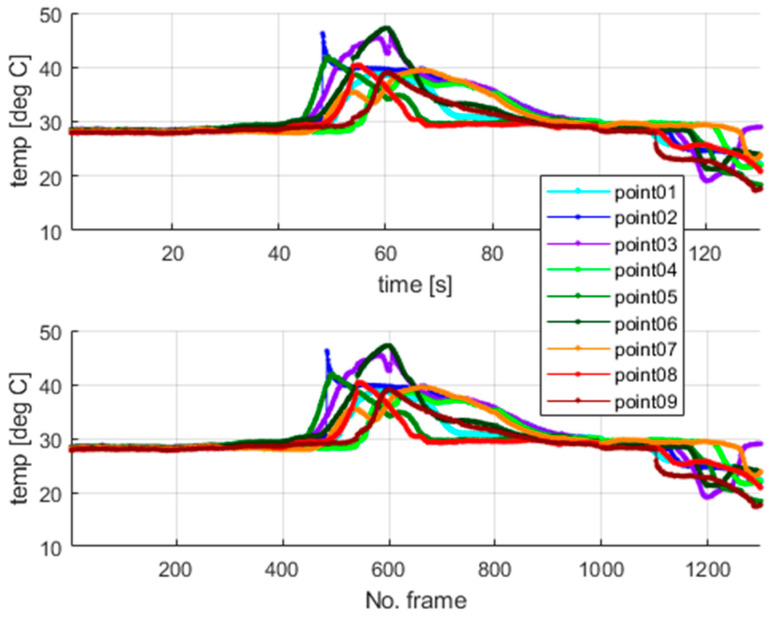
The temperature measured at the points marked in Figure 15.

**Figure 17:**
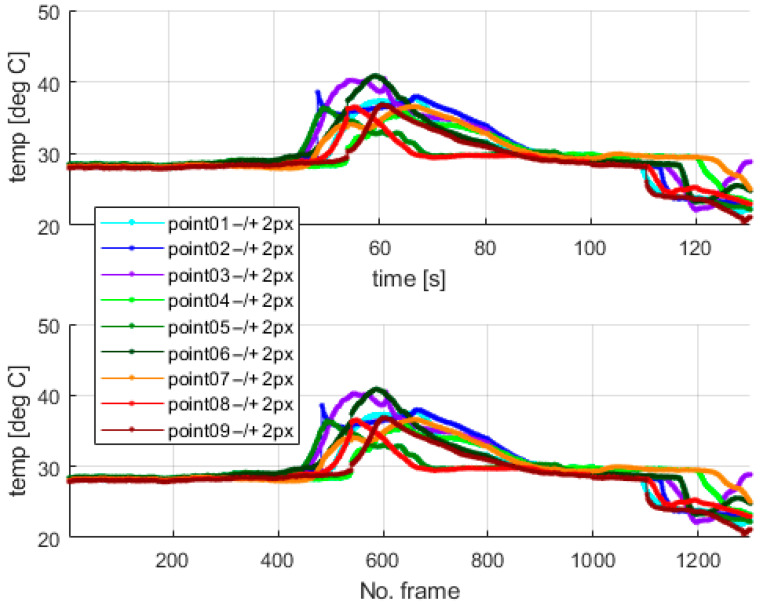
The average temperatures of areas of 5 × 5 pixel in the centers located at the points shown in Figure 15.

The plateau lines visible in Figure 14 are very smooth since the load rate is ten times greater than that assumed for the previous tests. Next, the thermovision data were analyzed. Figure 15 shows the selected points within the sample in the camera views. The temperature values for the selected points are presented in Figure 16. The calculated average temperatures for the square areas of 5 × 5 pixels, at the localizations shown in Figure 15, are presented in Figure 17.

As visualized in Figure 15, at the end of the test, i.e., beyond the 1300-th frame, the elongation force is too small to keep the wire straight, and the sample tries to recover its initial shape. Therefore, the thermal data are analyzed within the frame range from 1 to 1300. It should be mentioned that the thermal camera also recorded the results for the sections of the SMA wire which were covered by the extensometer assembly elements. The extensometer impacted the acquired results, and its influence could not be filtered out.

In the experiments with dedicated grips, more fluctuations regarding temperature courses are indicated, as shown in Figure 15, Figure 16 and Figure 17 (in comparison with Figure 10, Figure 11 and Figure 12). This observation confirms that more random, i.e., more spontaneous, material response is observed. This specific course of mechanically induced phase transition was expected and successfully confirmed by the measurements. Figure 15, Figure 16 and Figure 17 show that the order of occurrence of the temperature maxima is not maintained during the tensile test. Various shapes and amplitudes of the temporal temperature characteristics are indicated. The propagation of the temperature disturbance reflects the random nature of phase transformation. As observed, the clamping method considerably influences the material’s response. More uniform stress distribution can be achieved in the tested SMA sample. The more spontaneous course of phase transformation shows the usability of the proposed dedicated grips.

In the described test case, the use of a larger averaging window, i.e., the 5 × 5 pixel region (compared to the 3 × 3 pixel region applied in the case of measurements conducted for standard clamping jaws), allows for observation and interpretation of the measured temperature even though no clear piecewise monotonic courses are indicated in Figure 16 and Figure 17. The increase in the averaging region was necessary to identify the mechanically induced temperature change and track the progress of phase transformation. Propagation of the martensitic change front through the inspected sample could be observed as the stress gradually increased during the tensile test.

**Figure 18:**
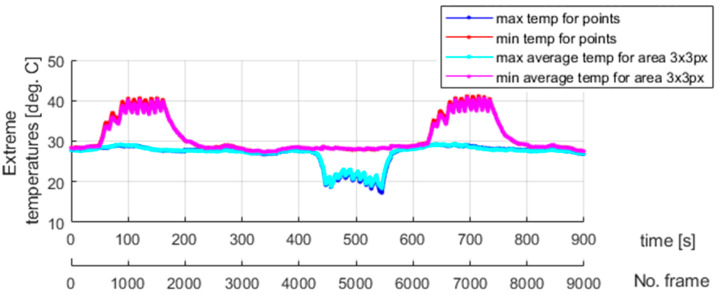
The extreme temperatures found based on the results of the second experiment shown in Figure 11.

**Figure 19:**
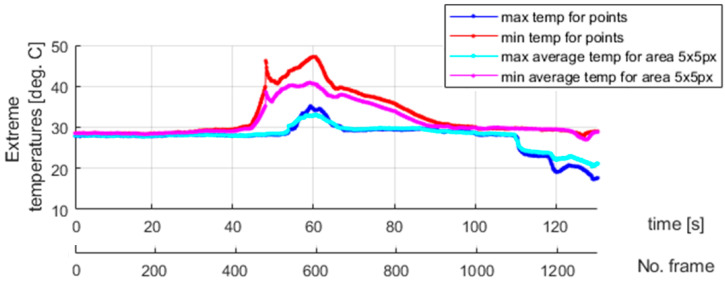
The extreme temperatures found based on the results of the third experiment shown in Figure 16.

Localization of the area where martensitic transformation is initiated is advantageously independent of the clamping positions of the tested sample. In other words, predicting where the phase changes begin is impossible. The transformation process is spontaneous. In this case, the observed behavior of an SMA material reflects the nature of its internal structure, in which the grain borders are randomly oriented. The use of microstructural models to study force interactions between grains and their importance for the material’s response is reported in [41,42,43]. The internal structure of an SMA sample is unknown a priori, and, therefore, the phenomenon mentioned above can be considered stochastic. To conclude, dedicated grips of special types, equipped with an extensometer, provide the means to assess the immanent randomness of the superelasticity effect. Finally, to allow for a comparison between the process dynamics, the extreme temperatures at the selected points for the second and third experiments are presented in Figure 18 and Figure 19, respectively.

Different dynamics of the martensitic transformation are identified for various grips used to clamp an SMA sample. As discussed in Section 4 and visualized in Figure 18, the phase transformation begins locally in a specific sample area for standard grips, which gradually changes its localization when the tensile test continues. In other words, no significant temperature change is observed elsewhere when heat is released or absorbed due to martensitic transformation in a given region. The temperature remains constant and equal to the ambient one. It is different in the case described in the present section. Figure 19 shows that there are time periods during which the martensitic transformation is observed in almost the entire investigated region of the tested sample. This phenomenon is more visible for the changes from the austenite to martensite phase—mainly during the 40 s long time interval caught approximately 40 s after data registration was started, as seen in Figure 19. Unfortunately, the assembly parts of the extensometer interfere with the temperature field registered in the thermograms. Notably, the mentioned issue affected the measurements carried out for reverse transformation. However, the uniform course of martensitic transformation for the entire sample, also exhibiting the same intensity for both the forward and reverse direction, can be easily recognized when studying the results shown in Figure 15 and Figure 19.

## 6. Summary and Final Conclusions

This paper discusses the clamping scheme’s influence on an SMA sample’s mechanical response during a tensile test. First, a tensile test was performed to identify the discrepancy between the experimental and simulation results. In this case, the conditions stated in [28] were satisfied. Therefore, the test was performed with a relatively low strain rate to avoid the influence of the endothermic and exothermic nature of the martensitic transformation. However, in the succeeding experiments, this restriction was neglected. Moreover, the strain rates were significantly increased. For the considered case, the impact of the exothermic and endothermic nature of the martensitic transformation was desired. The absorbed and released thermal energy could be successfully observed using a thermal camera. Therefore, the beginning and end of the martensitic transformation were correctly identified during tensile tests. However, comprehensive identification of the relationship between the clamping scheme-induced boundary conditions and the SMA response should be considered as going beyond the recent findings. The commonly applied standard [28] does not mention the risk of the existence of this correlation.

Confirmation of the main thesis stated by the authors regarding the influence of boundary conditions was achieved by performing two rounds of tensile tests—with standard grips (Section 4) and customized ones (Section 5). The results from both experiments were presented and preliminarily discussed in the respective sections. The main conclusions are given below.

The discrepancy between the results of the tensile test exhibiting a low strain rate (reported in Section 2) and the simulations (Section 3) is related to the assumption about 1D stress state simplification. The method used for calculating the stress value as the relation between tensile force and the cross-sectional area of the sample was not precise enough. Consequently, the application of the same type of load in the experiment and simulations may have led to differences in the results. The stress values, which were calculated for the simulations, are smaller than those identified during the experimental tests. According to the studies presented in Section 4 and Section 5, the reason for the phenomenon is omitting the additional load generated by the grips holding a specimen.

The subsequent experiments were performed with significantly increased strain rates. First, a test with the standard grips mounted was carried out. The analysis of temperatures recorded by the thermal camera shows that the stress fluctuation (stress front) in the sample propagates from the place where the material is held by the jaws towards the center of the SMA sample. This observation is related to the compression force generated by the fatigue machine at the gripper jaws. The authors consider that the martensitic transformation begins in the region where additional load is applied, even before the testing machine generates the tensile force. This configuration of the jaws induced specific boundary conditions, leading to the SMA wire’s non-uniform stress field. It should also be noted that the greatest stresses in the sample are expected to appear at the border between grains of the martensite and austenite phases, i.e., in the regions where the martensitic transformation occurs and propagates, as observed, primarily due to the grips’ impact. In this case, the compression force significantly influences the results of the tensile test.

The above-referenced phenomenon does not occur when dedicated grips are used. Following the concept proposed by the authors, the newly designed jaws were applied to decrease the value of local stresses related to clamping the SMA sample in the fatigue machine jaws. The results presented in Figure 19 prove that there is a specific time interval when the martensitic transformation is performed in the entire body of the specimen. This fact is related to a uniform stress field in the SMA sample during the tensile test. Therefore, the designed holders help to reduce maximal local stresses that are generated due to mounting a specimen in a fatigue testing machine. The change in the method of fixation of the SMA wire allows for the natural, stochastic characteristics of the martensitic transformation, which is visible in Figure 19. This behavior is not observed using standard grips. Consequently, the influence of the clamping scheme on the mechanical response of the SMA specimen using standard holders can be seen.

A considerably larger strain level was observed in the last experiment performed by the authors. This fact can be explained by the reduction in the significance of the multi-axial nature of a stress field when no additional transverse load is taken into account during a 1D tensile test—in contrast to the case of the compression force generated by standard jaws. The compression force applied by the standard holders could cause the generation of several martensite variants in the tested specimen. Their crystalline structures characterized various spatial orientations concerning the longitudinal axis of the SMA sample. Therefore, the martensitic transformation under a compression load did not cumulate strain in the direction of the tensile force. This fact led to a reduction in the total strain measured during the tensile test. However, more research needs to be conducted in the future to provide a better understanding of the observed phenomena.

In the present study, neither continuous stretching nor training processes were applied to indicate the repeatability of the SMA samples’ behavior regarding original shape recovery. However, a related conclusion can be drawn based on the experimental results for the examined SMA samples. An indirect auxiliary measure of original shape recovery can be formulated based on the stress–strain plots. This measure can describe the geometric similarity (repeatability) between the courses of the consecutive loops for the hysteretic stress–strain characteristics. The capability of original shape recovery can be assessed by analyzing Figure 4, Figure 7 and Figure 9, in which the pairs of plots for the subsequent tests are presented, e.g., the plots for the cases “test 1, loop 1”/“test 1, loop 2”, “test 2, loop 1”/“test 2, loop 2”, etc.

In the performed study, there was no negative interference regarding data interpretation for the actual scatter of the activation temperature, which was 10 °C ± 5 °C for the tested SMA samples, as stated in Section 2. However, a broader view of this material property and its standard deviation (or scatter) is worth being addressed. As confirmed in [44], the performance of SMA actuators highly depends on the alloy composition and the parameters of its postprocessing. It was confirmed that a slight change in the mentioned material’s composition could lead to considerable variation in the SMA sample’s transformation temperature. Consequently, any decrease in activation temperature repeatability may cause problems during experimental examination of the material’s mechanical response due to the greater contribution of randomness to phase transformation phenomena. This phenomenon’s expected less uniform course may negatively influence the inference of the tested sample properties. In other words, maintaining high precision regarding the SMA fabrication process ensures consistency and repeatability. The indicated specificity of SMAs certainly requires further research to be conducted.

The main conclusion drawn in this study is that the clamping method significantly influences the stress distribution in the tested SMA sample, which governs the course of phase transformation. The order of occurrence of the temperature maxima indicated during tensile tests confirmed the spontaneous nature of the martensitic transformation in the case of dedicated grips. Given the research that was conducted, however, new questions arise. The following issues require further study to be undertaken, advantageously pointing to interesting perspectives for future scientific works: (1) investigation of the kinetics and shape of the propagating phase transformation front, (2) investigation of the possibility of temporal suspension of the transformation process due to stress fluctuations, (3) formulation of the conditions for the uniform and random (spontaneous) course of the martensitic transformation, (4) analysis of the influence of the scatters of material parameters (including transformation temperatures) on the course of phase transformation and the shape of hysteretic stress–strain characteristics, and (5) more comprehensive study on the interaction between the type and directivity of crystalline structures and the resultant kinetics of the phase transformation.

## Figures and Tables

**Table 1:** Test schemes used to investigate phenomenon of superelasticity.

Steps	Course of Test			
	Test 1	Test 2	Test 3	Test 4
1. Pause period [s]	5	30	30	5
2. Displacement at stretching rate 0.05 mm/s [mm]	11	11	11	11
3. Pause period [s]	5	30	30	30
4. Displacement at relaxation rate 0.05 mm/s [mm]	−10.5	−11	−11	−11
5. Pause period [s]	-	30	30	30
6. Force at force rate 1 N/s [N]	10	10	10	-
7. Pause period [s]	5	5	5	30
Number of cycles for steps 1–6	2	2	2	1

**Table 2:** Material properties declared for FE model.

Parameter		Value
*E^A^*—Young’s modulus for austenite [GPa]		66.7
*E^S^*—Young’s modulus for martensite [GPa]		48.8
$\nu^{A}=\nu^{S}$—Poisson’s ratios [-]		$\left\{\begin{array}{c} 0\;\mathrm{for}\;1\text{-}D\;\mathrm{stress}\;\mathrm{field}\\ 0.3\;\mathrm{for}\;3\text{-}D\;\mathrm{stress}\;\mathrm{field} \end{array}\right.$
Characteristic stresses for the hysteretic stress–strain constitutive relation—referenced in Figure 5	$\sigma_{s}^{AS}$ [MPa]	600 ^1^ (530 ^2^)
	$\sigma_{f}^{AS}$ [MPa]	630 ^1^ (595 ^2^)
	$\sigma_{s}^{SA}$ [MPa]	420 ^1^ (185 ^2^)
	$\sigma_{f}^{SA}$ [MPa]	350 ^1^ (145 ^2^)
ε—transformation strain [-]		0.075

^1^ The discussion of the overestimated values applied for FE simulations is found in the current section. ^2^ The experimentally identified values are referenced in Figure 5.

**Table 3:** Experimental test schemes used to investigate second SMA sample.

Steps	Course of Test	
	Test 1	Test 2
1. Pause period [s]	5	5
2. Displacement at stretching rate 0.05 mm/s [mm]	11	13
3. Pause period [s]	30	30
4. Displacement at relaxation rate 0.05 mm/s [mm]	-	−13
5. Pause period [s]	-	5
6. Force at force rate 1 N/s [N]	10	10
7. Pause period [s]	5	5
Number of cycles for steps 1–6	1	2

**Table 4:** Experimental test scheme used to investigate third SMA sample.

Steps	Course of Test
1. Pause period [s]	5
2. Displacement at stretching rate 0.5 mm/s [mm]	30
3. Pause period [s]	5
4. Displacement at relaxation rate 0.5 mm/s [mm]	−30
5. Pause period [s]	5
Number of cycles for steps 1–5	1

## Data Availability

The original contributions presented in this study are included in the article. Further inquiries can be directed to the corresponding author.

## Abbreviations

The following abbreviations are used in this manuscript:

1-D	One-dimensional
3-D	Three-dimensional
DSC	Differential scanning calorimetry
FE	Finite element
SMA	Shape memory alloy
SMM	Shape memory material
SMP	Shape memory polymer
